# Inhibition of Acyl-CoA Synthetase Long-Chain Family Member 4 Facilitates Neurological Recovery After Stroke by Regulation Ferroptosis

**DOI:** 10.3389/fncel.2021.632354

**Published:** 2021-04-06

**Authors:** Junmin Chen, Lan Yang, Lianxia Geng, Junna He, Lei Chen, Qian Sun, Jing Zhao, Xiaopeng Wang

**Affiliations:** Second Hospital of Hebei Medical University, Shijiazhuang, China

**Keywords:** cerebral ischemia, ferroptosis, GPx4, ACSL4, neurological recovery

## Abstract

**Background:**

Ischemic stroke is the main cause of disability worldwide, leading to a serious socioeconomic burden. Ferroptosis is a non-apoptotic form of programmed cell death and is related to various diseases. Acyl-CoA synthetase long-chain family member 4 (ACSL4) is considered a target of ferroptosis, but its specific role in ischemic stroke remains unclear. In this study, we investigate whether the inhibition of ACSL4 promotes the recovery of neurological function in a way that prevents ferroptosis.

**Methods:**

A transient cerebral ischemia model was established for mice by middle cerebral artery occlusion (MCAO); glutathione peroxidase 4 (GPx4), ACSL4 and cyclooxygenase 2 (COX2) were detected by Western blot, and changes to mitochondria were observed by a transmission electron microscope. A kit was used to determine iron levels and lipid peroxide indicators, such as glutathione peroxidase (GPx), reduced glutathione (GSH), total glutathione/oxidized glutathione (GSH/GSSG), lipid peroxidation, reactive oxygen species, superoxide and malonaldehyde. Following MCAO, a ferroptosis inhibitor, liproxstatin-1, was administered intranasally immediately at a concentration of 10 mg/kg. Rosiglitazone was used to inhibit ACSL4 and was administered intravenously 1 h before MCAO at a concentration of 0.4 mg/kg. Brain injury was determined by neurological deficit scores, neuroscore (28-point), corner test and gait analyses, at 24 and 72 h after stroke. Brain infarct volume was determined by 2, 3, 5-Triphenyltetrazolium chloride (TTC) staining at 72 h after stroke.

**Results:**

After MCAO, GPx4 protein expression decreased, ACSL4 and COX2 protein expression increased, GPx activity decreased and iron accumulation. Transmission electron microscopy confirmed that the outer mitochondrial membrane of neurons had ruptured and mitochondrial cristae had decreased or disappeared. Liproxstatin-1 could significantly attenuate the decrease of GPx4 and the increase of COX2 after MCAO, dramatically reducing iron accumulation and decreasing GPx activity, accompanied by a marked reduction in changes in lipid peroxidation indicators. The use of rosiglitazone to inhibit ACSL4 could significantly improve neurological function and reduce the brain infarct volume at 72 h after stroke. Importantly, inhibiting ACSL4 could significantly attenuate the decline of GPx4 after MCAO and markedly attenuate iron accumulation and a decrease in GPx activity. Additionally, changes in lipid peroxidation indicators were also significantly inhibited.

**Conclusion:**

This study indicates that inhibiting ACSL4 can promote the recovery of neurological function after stroke by suppression of ferroptosis.

## Introduction

Ischemic stroke is a leading cause of death and disability worldwide, causing severe health and socioeconomic burdens (Benjamin et al., 2019). Ischemic stroke is triggered by reduced or interrupted blood flow, resulting in insufficient oxygen and glucose supply and eventually brain injury (Barrington et al., 2017). Intravenous thrombolysis and endovascular therapy have been used for clinical intervention. However, there are still a large number of patients with residual neurological deficits due to a narrow time window and side-effects (Powers et al., 2019). Regulating the pathogenesis of brain injury after stroke has established roles for multiple processes including oxidative stress, inflammation, excitotoxicity and apoptosis (Tuo et al., 2017). Recently, ferroptosis, a new form of cell death related to oxidative stress, has attracted increasing attention. Ferroptosis appears in a variety of diseases, such as Alzheimer’s disease, ischemic stroke, intracerebral hemorrhage, tumors and heat stress in plants (Tuo et al., 2017; Alim et al., 2019; Li et al., 2019). Therefore, comprehensively elucidating the molecular mechanism of ferroptosis in ischemic stroke and finding key signal pathways and protein molecules for ferroptosis are of great significance in terms of guiding the clinical practice of ischemic stroke (Dong et al., 2018).

Ferroptosis is a non-apoptotic form of programmed cell death characterized by an iron-dependent accumulation of reactive oxygen species (ROS) (Lei et al., 2020). Ferroptosis can be induced by the small molecule erastin, which inhibits cystine import, causing glutathione depletion and inactivation of the glutathione peroxidase 4 (GPx4). Inactivation of GPx4 cannot inhibit the production of ROS, resulting in the accumulation of lipid peroxidation and ultimately cell death (Dixon et al., 2012; Stockwell et al., 2017; Alim et al., 2019). Lipid peroxidation in ferroptosis involves preferential oxidation of phosphatidylethanolamine (PE) containing arachidonic acid (AA) and adrenic acid (AdA) (Angeli et al., 2017), both of which are types of polyunsaturated fatty acids (PUFAs). During the ferroptosis process, PUFAs can be oxidized to metabolites, e.g., hydroxyeicosatetraenoic acids (HETEs) (Yang et al., 2016; Angeli et al., 2017; Li et al., 2019). Cyclooxygenase 2 (COX2) is the key enzyme in the initial step of catalyzing the synthesis of prostaglandin (PG) from AA in the body. Cyclooxygenase 2 can be used as a biomarker of ferroptosis (Chen B. et al., 2019). Although reports of ferroptosis in ischemic stroke have been reported (Tuo et al., 2017), the underlying mechanism remains unclear.

Acyl-CoA synthetase long-chain family member 4 (ACSL4), an enzyme involved in the activation of PUFAs, has been found to facilitate the esterification of AA and adrenoyl into PE, which is a process closely related to ferroptosis (Yang and Stockwell, 2016; Angeli et al., 2017). A recent report suggests that ACSL4 mediated production of 5-HETE contributed to ferroptosis (Yuan et al., 2016). Moreover, genetic or pharmacological inactivation of ACSL4 is refractory to ferroptosis, suggesting that ACSL4 may be a target for ferroptosis inhibition (Angeli et al., 2017; Doll et al., 2017; Li et al., 2019). Although ACSL4 is considered a sensitive monitor and an important contributor to ferroptosis (Yuan et al., 2016), its specific role in ischemic stroke is unknown. Hence, there is a need for better understanding the function and significance of ACSL4 in ischemic stroke and its relationship with ferroptosis.

In the current study, we speculate that ferroptosis may be an important mechanism of ischemic stroke and that the inhibition of ferroptosis is beneficial to neurological recovery after ischemic stroke. Meanwhile, we sought to examine the efficacy of ACSL4 on modulating ferroptosis in a cerebral ischemia model and focused on whether inhibition of ACSL4 facilitated neurological recovery in an anti-ferroptosis manner.

## Materials and Methods

### Experimental Animals

Male C57BL/6 mice (age 8–10 weeks, weight 23–25 g, RRID: IMSR_CRL:27) of specific-pathogen-free grade were purchased from the Vital River Laboratory Animal Technology Co., Ltd (Beijing, China). All animals were kept on a 12-h light/dark cycle with controlled humidity (60% ± 5%) and temperature (22°C ± 3°C) and had free access to food and water. All animals were allowed to acclimatize to the new surroundings for at least 3 days before use in experiments. Animals were assigned randomly using a random number table. All assessments were conducted by investigators who were blinded to experimental group assignment. All experimental procedures were approved by the Committee of Experimental Animals of Hebei Medical University (Shijiazhuang, China) and conducted in accordance with the National Institutes of Health guidelines. Sample sizes were not estimated prior to conducting experiments. Rather, animals were allocated into each group until sample sizes were similar to that of other MCAO studies. The mice were randomly divided using a random number table generated by SPSS software (v. 21.0; SPSS, RRID:SCR_002865) (Zhao et al., 2015).

### Transient Cerebral Ischemia by Middle Cerebral Artery Occlusion

Mice were fixed and anesthetized by intraperitoneal (i.p.) injection of 1% pentobarbital (100 mg/kg). Transient focal cerebral ischemia was produced in the right middle cerebral artery occlusion (MCAO) using the intraluminal filament technique. The monofilament was withdrawn after 1 h to restore blood flow. The right carotid artery was exposed to dissect the external carotid arteries (ECA) and the internal carotid arteries (ICA), blocking the ECA at the level of the middle cerebral artery (MCA) branch. A monofilament nylon suture (Yushun Biotech, Guangzhou, China) with a rounded tip was inserted through the ICA. When slight resistance was felt, threading was stopped and fixed. After 1 h, the filament was gently removed for reperfusion. The mock-operated control mice underwent the same steps except for inserting the filament. Before, during and after MCAO, the local cerebral blood flow was monitored by laser Doppler flowmeter (Moor VMS-LDF, Moor Instruments Ltd., Axminster, Devon, United Kingdom), and the MCA was occluded based on a criterion of *a* < 25% baseline blood flow remaining after MCAO. A constant temperature heating pad at 37°C was used during and after the operation to maintain the temperature of the mouse at 37 ± 0.5.

### Inhibitor Treatment and Experimental Design

Liproxstatin-1 (Lip, MedChemExpress Cat#HY-10108), a ferroptosis inhibitor, was administered intranasally immediately after MCAO at a concentration of 10 mg/kg (Tuo et al., 2017). Rosiglitazone (ROSI, Selleck Cat#S2556) is used to inhibit ACSL4, a classic peroxisome proliferator-activated receptor-γ agonist. In the current study, it which was administered intravenously 1 h before MCAO at a concentration of 0.4 mg/kg. The mice were randomly divided using a random number table generated by SPSS software. In preliminary experiments, mice were randomly assigned into two groups: the sham group (mice receiving a mock operation) and the MCAO group (mice received MCAO). In subsequent experiments, mice were randomly assigned into four groups: sham group, MCAO group, sham + Lip group (mice receiving mock treatment and intranasally liproxstatin-1) and MCAO + Lip group (mice receiving MCAO and intranasally liproxstatin-1). Additionally, mice were randomly assigned into four groups: sham, MCAO, sham + ROSI (mice receiving a mock operation and intravenously rosiglitazone) and MCAO + ROSI groups (mice receiving MCAO and intravenously rosiglitazone). According to the experimental design, the mice were quickly sacrificed under deep anesthesia and samples were collected for further study.

### Neurobehavioral Tests

CatWalk XT (v10.6, Noldus Information Technology), a gait analysis system, was used to measure footfalls and motor performance in ischemic mice as described in Caballero-Garrido et al. (2017). Each mouse walked freely through a corridor on a glass sidewalk that was illuminated from below. A camera was used to collect the movements of all four paws at a rate of 100 frames/sec for 20 s; CatWalk XT software was then used for analysis. Walking at a steady speed (without stopping, grooming, or rearing) was considered a successful walking test. Prior to the MCAO, the mice in each group were continuously trained for 3 days. For the test, mice were placed on a glass sidewalk with a speed of 8 cm/s or the maximum speed at which mice could maintain optimally coordinated locomotion. Background images were recorded prior to each test day. Each mouse was performed three times, with an interval of more than 15 min each time. Instantaneous running speed (mm/s), stride-length of the left front limb (mm) and the print area of the left front limb (mm^2^) were selected for gait analysis.

Neurological deficit scores were derived via a neurological test, which was performed by a blinded examiner prior to, 24 and 72 h after MCAO as previously described (Xue et al., 2019). In short, 0 represents no deficit; 1 represents difficulty fully extending the contralateral forelimb; 2 represents an inability to extend the contralateral forelimb; 3 represents gentle circling to the contralateral forelimb; 4 represents severe circling movements; 5 represents falling to the contralateral side. The higher the neurological deficit score, the more severe the impairment of motor function.

Neuroscore (28-point) was used to estimate sensorimotor function before, 24 and 72 h after MCAO as previously described (Encarnacion et al., 2011). This neuroscore used 11 tests with a cumulative maximum score of 28 points: (1) circling (maximum 4); (2) motility (maximum 3); (3) general condition (maximum 3); (4) righting reflex when placed on back (maximum 1); (5) paw placement of each paw onto a tabletop (maximum 4); (6) behavior on a horizontal bar (maximum 3); (7) behavior on an inclined platform (maximum 3); (8) grip strength (maximum 2); (9) contralateral reflex (maximum 1); (10) contralateral rotation (maximum 2); (11) visual forepaw reaching (maximum 2). The lower the neuroscore, the more severe the impairment of sensorimotor function.

A corner test was performed to estimate sensorimotor asymmetry as previously described (Xue et al., 2019). The experimental device includes two boards at an angle of 30°, with a small gap between the two boards intended to encourage mice to enter into. When mice entered the corner, the vibrissae on both sides will touch the board and be stimulated. At this time, the mouse will move forward or upward and then turn 180° to face the open end of the angle. The probability of normal mice turning left or right is nearly equal; however, following MCAO, mice preferentially turn to the ipsilateral injury. The test was repeated 10 times with a minimum interval of 30 s between trials. Subsequently, the number of right turns was counted.

### Measurement of Infarction Volume

2,3,5-Triphenyltetrazolium chloride (TTC) staining was performed to evaluate the infarct volume at 72 h after reperfusion. Brain tissue was sliced into seven coronal sections (1mm thick), stained with a 2% solution of TTC. Percentage hemisphereisphere lesion volume = {[total infarct volume - (volume of intact ipsilateral hemisphere - volume of intact contralateral hemisphere)]/contralateral hemisphere volume}.

### 2-Dimensional Laser Speckle Imaging Techniques

The cortical cerebral blood flow (CBF) was monitored by a 2-D laser speckle imager (PeriCam PSI, Sweden). laser speckle perfusion images were acquired before middle cerebral artery occlusion, during ischemia, and after reperfusion. Cerebral blood flow is expressed as a percentage of pre-MCAO baselines (Liu et al., 2016; Xue et al., 2019).

### Western Blot

Cortical tissue was extracted from the peri-infarct area 24 h after MCAO and from the corresponding area of the mock-operated mice. Protein from cortical tissue was extracted using a total protein extraction kit (Applygen Cat#P1250) with a 2% protease inhibitor cocktail (Sigma Cat# P8340) and a 1% phosphatase inhibitor (Applygen Cat#P1260), according to the description in Chen J. et al. (2019), and the entire extraction process was performed on ice or at 4°C. The protein concentration of the tissue sample was measured using a bicinchoninic acid (BCA) protein assay reagent kit (Thermo Scientific, United States). An equivalent amount of protein (50 μg) was separated by a 12% sodium dodecyl sulfate–polyacrylamide gel electrophoresis and transferred to polyvinylidene fluoride (PVDF) membranes (Roche, United States). The PVDF membranes were then blocked with 5% non-fat milk powder at room temperature for 1 h and incubated with the primary antibodies overnight at 4°C. Primary antibodies included GPx4 (1:5000, Abcam Cat#ab125066, RRID: AB_10973901), ACSL4 (1:1000, Abcam Cat# ab155282, RRID: AB_2714020), COX2 (1:800, Abcam Cat#ab62331, RRID: AB_942121) and GAPDH (1:10000, Bioworld Technology Cat#AP0063, RRID: AB_2651132). On the second day, the membranes were incubated with the fluorescent labeling secondary antibody (Goat Anti-Rabbit IgG Antibody DyLight 800 Conjugated, 1:10000, Rockland Cat#611-145-122, RRID: AB_1057618) for 1 h at room temperature. The relative density of each band was detected by an Odyssey infrared scanner (LI-COR Bioscience, United States). The intensity of the bands was analyzed by Image J software. The GAPDH antibodies were used for normalization.

### Transmission Electron Microscopy

Mice were deeply anesthetized and perfused with precooled phosphate-buffered saline (PBS, pH 7.4) and 2.5% glutaraldehyde (Servicebio Cat#G1102). A 1-mm-cube tissue block was obtained from the peri-infarct area 24 h after MCAO and from the corresponding area of the mock-operated mice. The tissue blocks were then steeped in the 2.5% glutaraldehyde at 4°C for 2–4 h. The samples were post-fixed with 1% osmium tetroxide in a 0.1 M phosphate buffer (PB, pH 7.4) at room temperature for 2 h, dehydrated in a graded ethyl alcohol series, and embedded in SPI-Pon 812 epoxy resin (SPI Cat#90529-77-4) overnight at 37°C. The polymerization was performed at 60°C for 48 h. Ultrathin sections (60–80 nm) were cut using a Leica UC7 ultramicrotome, stained with 2% uranyl acetate and lead citrate for 15 min, and viewed under an HT7700 transmission electron microscope (TEM, HITACHI, Japan).

### Iron Assay

Fresh tissue samples (50 mg) from the peri-infarct area 24 h after MCAO and from the corresponding area of the mock-operated mice were immediately homogenized with precooled PBS; then, the supernatant was collected by centrifugation. The iron level was measured using an Iron Assay Kit (Abcam Cat#ab83366), according to the manufacturer’s instructions.

### Glutathione Peroxidase 4 Activity Assay

Fresh tissue samples (50 mg) were collected from the peri-infarct area at 24 h after MCAO and from the corresponding area of the mock-operated mice and then performed into a homogenate (concentration 1%–10%) with precooled PBS. The GPx activity was analyzed using the Glutathione Peroxidase Assay Kit (Abcam Cat# ab102530), according to the manufacturer’s instructions.

### Reduced Glutathione and Total Glutathione/Oxidized Glutathione Assays

Tissue samples were prepared as described above. Reduced glutathione (GSH) was analyzed using the Reduced Glutathione Assay Kit (Nanjing Jiancheng Bioengineering Institute Cat#A006, China), according to the manufacturer’s instructions. Total glutathione/oxidized glutathione (GSH/GSSG) was determined using the Total Glutathione/Oxidized Glutathione Assay Kit (Nanjing Jiancheng Bioengineering Institute Cat#A061, China), following the manufacturer’s instructions.

### Superoxide Dismutase and Malondialdehyde Assays

Tissue samples were prepared as described above. Superoxide dismutase (SOD) was analyzed using the Superoxide Dismutase Assay Kit (Nanjing Jiancheng Bioengineering Institute Cat#A001, China), according to the manufacturer’s instructions. The results were evaluated using units per microgram of total protein (U/mg prot) and the protein concentration was measured using a BCA protein assay reagent kit (Thermo Scientific, United States). Malonaldehyde (MDA) was determined using the Malondialdehyde Assay Kit (Nanjing Jiancheng Bioengineering Institute Cat#A003, China), following the manufacturer’s instructions. The result was evaluated using nanomoles per microgram of total protein (nmol/mg prot) and the protein concentration was measured using a BCA protein assay reagent kit (Thermo Scientific, United States).

### Lipid Peroxidation Assay

Tissue samples were prepared as described above. A lipid peroxidation assay (LPO) kit (Nanjing Jiancheng Bioengineering Institute Cat#A106, China) was used to test the LPO level in lysates of brain tissue of the peri-infarct area at 24 h after MCAO and the corresponding area of the mock-operated mice, following the manufacturer’s protocol. The results were evaluated using micromoles per gram of total protein (μmol/g prot) and the protein concentration was measured using a BCA protein assay reagent kit (Thermo Scientific, United States).

### Reactive Oxygen Species Assay

Tissue samples were prepared as described above. A reactive oxygen species assay kit (Nanjing Jiancheng Bioengineering Institute Cat#E004, China) was used to test the ROS level in lysates of brain tissue of the peri-infarct area at 24 h after MCAO and the corresponding area of the mock-operated mice, following the manufacturer’s protocol.

### Statistical Analysis

Statistical analysis was performed using SPSS software (v. 21.0; Version X, IBM, United States). The results were expressed as the mean ± standard deviation (SD) if normally distributed; otherwise, they were expressed as median ± interquartile range. Comparisons between two groups were performed by Student’s *t*-test. Statistical comparisons between multiple groups were evaluated using one-way analysis of variance (ANOVA) followed by the least significant difference (LSD) test. Non-parametric analyses were performed using the Mann–Whitney U test for data that were not normally distributed (neurological deficit scores). A two-sided value of *P* < 0.05 was considered statistically significant. Curve fitting was obtained using GraphPad Prism software (v. 8.0.2; GraphPad Software, RRID: SCR_002798).

## Results

### Ferroptosis Does Occur After Stroke

A total of 29 mice died before completion of the experiment and were excluded from the study: Eighteen mice (14.29%) in MCAO group, four mice (9.09%) in the MCAO + Lip group and seven mice (10.45%) in MCAO + ROSI group. After anatomy, no brain or subarachnoid hemorrhage was found in these animals, and a small number of animals had an excessively large infarct area.

**FIGURE 1:**
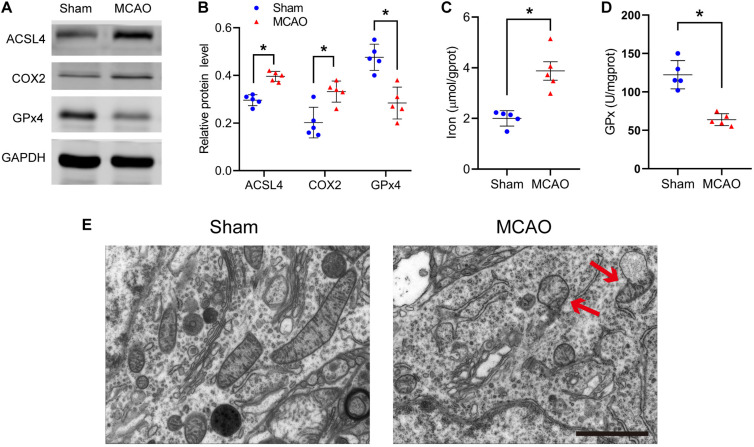
Ferroptosis does occur after stroke. **(A)** The protein levels of ACSL4, COX2, and GPx4 in peri-infarct region of sham group and MCAO group at 24 h after cerebral ischemic reperfusion, respectively. **(B)** Quantification of ACSL4, COX2, and GPx4 relative expression in mice. *P < 0.05, *n* = 5 each group. **(C,D)** Fresh tissue samples from the peri-infarct area at 24 h after stroke were collected to measure iron and GPx4 activity. *P < 0.05, *n* = 5 each group. **(E)** Representative Transmission electron microscopy images in peri-infarct region of sham group and MCAO group at 24 h after cerebral ischemic reperfusion are shown. The red arrows indicate the outer mitochondrial membrane of neurons was ruptured and the mitochondrial cristae decreased or disappeared. Bar = 2 μm.

After stroke, the key protein of ferroptosis, GPx4, decreased significantly (sham vs MCAO group: 0.48 ± 0.05 vs 0.28 ± 0.07, *p* ≤ 0.05; *n* = 5, Student’s *t*-test, Figures 1A,B). Compared with the sham group, MCAO significantly increased the protein levels of COX2 in mice (0.20 ± 0.06 vs 0.33 ± 0.04, *p* ≤ 0.05; *n* = 5, Student’s *t*-test, Figures 1A,B). Similarly, compared with the sham group, the MCAO group significantly increased the ACSL4 protein levels of mice (0.30 ± 0.02 vs 0.40 ± 0.02, *p* ≤ 0.05; *n* = 5, Student’s *t*-test, Figures 1A,B), suggesting that ACSL4 may serve as a key target after cerebral infarction.

Concurrently, we found that iron, another indispensable factor for the execution ferroptosis, was significantly elevated after ischemia (2.00 ± 0.31 vs 3.87 ± 0.81 *p* ≤ 0.05; *n* = 5, Student’s *t*-test, Figure 1C). Similar to the western blot results, GPx activity was significantly decreased after ischemia (122.33 ± 18.36 vs 63.77 ± 7.76, *p* ≤ 0.05; *n* = 5, Student’s *t*-test, Figure 1D).

Transmission electron microscopy was used to study the morphological features of ferroptosis. We observed that the outer mitochondrial membrane of neurons had ruptured and the mitochondrial cristae had decreased or disappeared (Figure 1E). The above results all suggest that ferroptosis does occur after cerebral infarction.

### Liproxstatin-1 Inhibits Ferroptosis After Stroke

Liproxstatin-1 is an effective and specific ferroptosis inhibitor that has previously been shown to limit the ischemia -reperfusion injury of brain tissue (Tuo et al., 2017).

**FIGURE 2:**
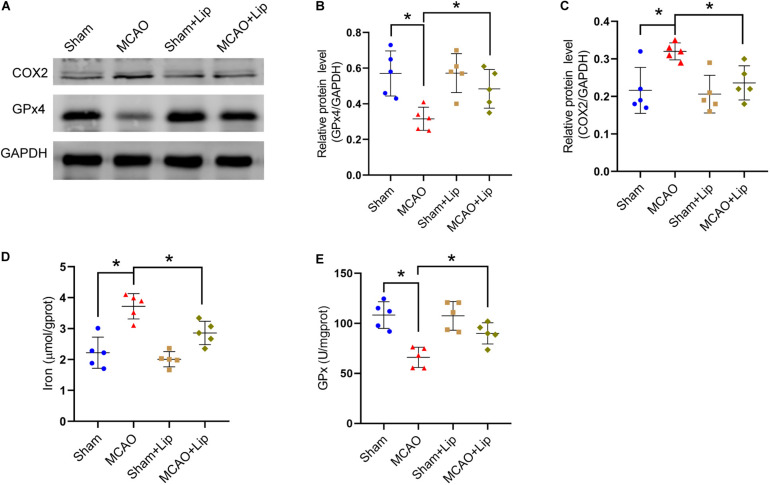
Liproxstatin-1 inhibits ferroptosis after stroke. **(A)** The protein levels of COX2 and GPx4 in peri-infarct region of sham group, MCAO group, sham + Lip group and MCAO + Lip group at 24 h after cerebral ischemic reperfusion, respectively. **(B,C)** Quantification of GPx4 and COX2 relative expression in mice. *P < 0.05, *n* = 5 each group. **(D,E)** Fresh tissue samples from the peri-infarct area of sham group, MCAO group, sham + Lip group and MCAO + Lip group at 24 h after stroke were collected to measure iron and GPx4 activity. *P < 0.05, *n* = 5 each group.

Compared with the MCAO group, MCAO with liproxstatin-1 treatment significantly attenuated the decrease in the protein level of GPx4 in mice caused by stroke (MCAO vs MCAO + Lip group: 0.32 ± 0.06 vs 0.48 ± 0.11, *p* ≤ 0.05; *n* = 5, one-way ANOVA, Figures 2A,B). Similarly, compared with the MCAO group, MCAO with liproxstatin-1 treatment significantly attenuated the increase in the protein level of COX2 in mice caused by stroke (0.32 ± 0.02 vs 0.24 ± 0.05, *p* ≤ 0.05; *n* = 5, one-way ANOVA, Figures 2A,C).

Moreover, we found that MCAO with liproxstatin-1 treatment significantly attenuated the iron accumulation caused by stroke, compared with the MCAO group (3.72 ± 0.41 vs 2.86 ± 0.38, *p* ≤ 0.05; *n* = 5, one-way ANOVA, Figure 2D). Compared with the MCAO group, GPx activity was significantly increased in the MCAO + Lip group (66.03 ± 10.14 vs 89.97 ± 10.58, *p* ≤ 0.05; *n* = 5, one-way ANOVA, Figure 2E).

### Inhibition of Ferroptosis Attenuate Lipid Peroxidation After Stroke

**FIGURE 3:**
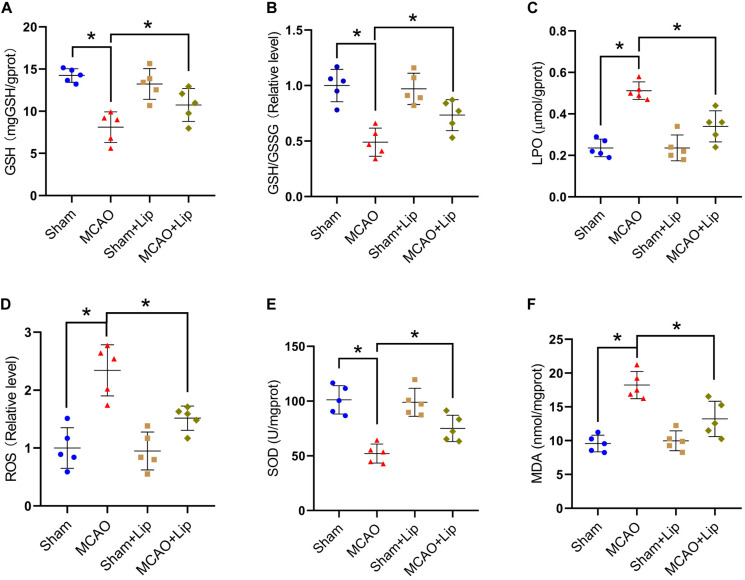
Inhibition of ferroptosis attenuate oxidative stress after stroke. Fresh tissue samples from the peri-infarct area of sham group, MCAO group, sham + Lip group and MCAO + Lip group at 24 h after stroke were collected to measure GSH level **(A)**, the GSH/GSSG ratio **(B)**, LPO level **(C)**, the ROS ratio **(D)**, SOD level **(E)**, and MDA level **(F)**. *P < 0.05, *n* = 5 each group.

Previous studies posited that ferroptosis is related to lipid peroxidation. We further examined the changes in lipid peroxidation indicators after stroke. Compared with the sham group, the MCAO group had significantly decreased the level of GSH and the relative level of GSH/GSSG (GSH: 14.24 ± 0.81 vs 8.11 ± 1.82, GSH/GSSG: 1.00 ± 0.16 vs 0.49 ± 0.13, *p* ≤ 0.05; *n* = 5, one-way ANOVA, Figures 3A,B). Compared with the MCAO group, MCAO with liproxstatin-1 treatment significantly attenuated the decrease in the level of GSH and the relative level of GSH/GSSG in mice caused by stroke (GSH: 8.11 ± 1.82 vs 10.74 ± 1.95, GSH/GSSG: 0.49 ± 0.13 vs 0.73 ± 0.14, *p* ≤ 0.05; *n* = 5, one-way ANOVA, Figures 3A,B).

Compared with the sham group, MCAO significantly increased the levels of LPO and the relative level of ROS (LPO: 0.23 ± 0.04 vs 0.51 ± 0.14, ROS: 1.00 ± 0.35 vs 2.34 ± 0.44, *p* ≤ 0.05; *n* = 5, one-way ANOVA, Figures 3C,D). Compared with the MCAO group, the levels of LPO and the relative level of ROS were significantly decreased in the MCAO + Lip group (LPO: 0.51 ± 0.14 vs 0.34 ± 0.07, ROS: 2.34 ± 0.44 vs 1.52 ± 0.21, *p* ≤ 0.05; *n* = 5, one-way ANOVA, Figures 3C,D).

The SOD level was significantly decreased after ischemia (101.16 ± 12.93 vs 52.10 ± 8.70, *p* ≤ 0.05; *n* = 5, one-way ANOVA, Figure 3E). The MDA level was significantly increased after ischemia (9.57 ± 1.23 vs 18.23 ± 2.02, *p* ≤ 0.05; *n* = 5, one-way ANOVA, Figure 3F). Compared with the MCAO group, MCAO with liproxstatin-1 treatment significantly attenuated the decrease of SOD level and the increased MDA level in mice caused by stroke (SOD: 52.10 ± 8.70 vs 75.00 ± 11.97, MDA: 18.23 ± 2.02 vs 13.23 ± 2.62, *p* ≤ 0.05; *n* = 5, one-way ANOVA, Figures 3E,F). The above results indicate that ferroptosis after stroke was related to lipid peroxidation and inhibiting ferroptosis could reduce lipid peroxidation.

### Inhibition of ACSL4 Ameliorate Post-stroke Neurological Function and Infarct Volume

A CatWalk test, neurological deficit scores, neuroscore (28-point) and a corner test were selected to assess the neurological function of ischemic injury.

**FIGURE 4:**
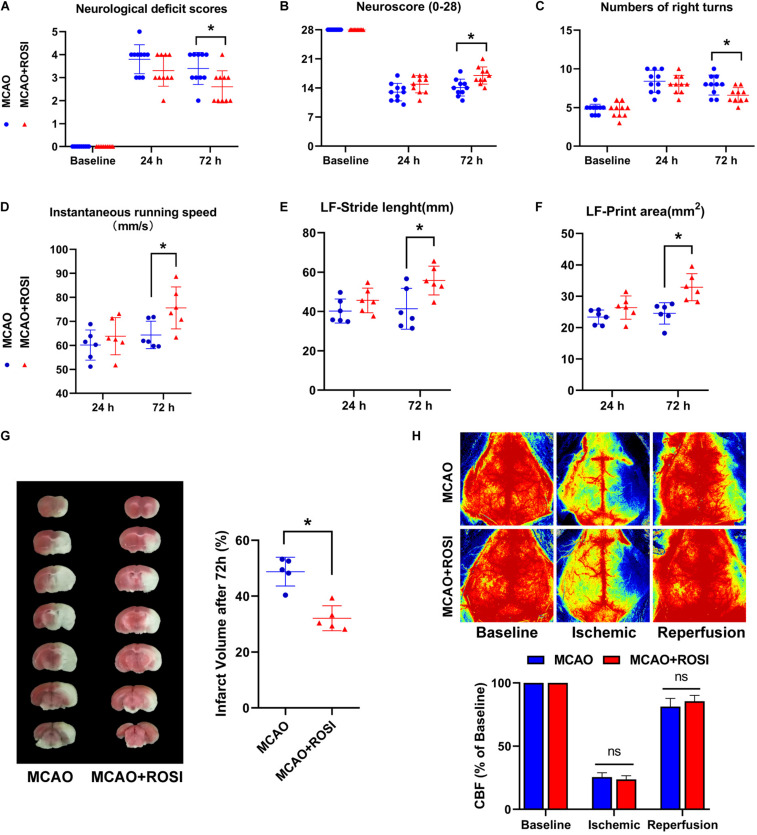
Inhibition of ACSL4 ameliorate post-stroke neurological function and infarct volume. **(A)** Neurological deficits scores of MCAO and MCAO + ROSI mice before and at 24 and 72 h after cerebral ischemic reperfusion. *P < 0.05, *n* = 10 each group. **(B)** Neuroscore (28-point) of MCAO and MCAO + ROSI mice before and at 24 and 72 h after stroke. *P < 0.05, *n* = 10 each group. **(C)** Corner test of MCAO and MCAO + ROSI mice before and at 24 and 72 h after stroke. *P < 0.05, *n* = 10 each group. **(D)** Instantaneous running speed of MCAO and MCAO + ROSI mice before and at 24 and 72 h after stroke. *P < 0.05, *n* = 6 each group. **(E)** The stride length for left front limb of MCAO and MCAO + ROSI mice before and at 24 and 72 h after stroke. *P < 0.05, *n* = 6 each group. **(F)** The print area for left front limb of MCAO and MCAO + ROSI mice before and at 24 and 72 h after stroke. *P < 0.05, *n* = 6 each group. **(G)** Representative TTC stained sections of MCAO and MCAO + ROSI mice at 72 h after stroke. *P < 0.05, *n* = 5 each group. **(H)** Representative 2-D laser speckle images of MCAO and MCAO + ROSI mice. *n* = 5 each group.

The results of neurological deficits scores indicated that neurological deficits were significantly improved in MCAO + ROSI mice compared with MCAO mice 72 h after ischemia, which suggested restoration of neurological functions (MCAO vs MCAO + ROSI group: 3.50 ± 1.00 vs 2.50 ± 1.00, *p* ≤ 0.05; *n* = 10, Mann–Whitney U test, Figure 4A). However, there was no statistical significance between the MCAO group and MCAO + ROSI group at 24 h after ischemia.

The (28-point) neuroscore exhibited a general neurological deficit at 24 and 72 h after MCAO compared with the baseline. Importantly, MCAO mice exhibited significant impairment compared with MCAO + ROSI mice 72 h after ischemia (14.10 ± 2.02 vs 17.00 ± 2.05, *p* ≤ 0.05; *n* = 10, Student’s *t*-test, Figure 4B).

In the corner test, mice that received MCAO indicated an increased number of right turns compared to the baseline. Moreover, MCAO + ROSI mice showed fewer right turns compared with MCAO mice 72 h after MCAO (7.90 ± 1.29 vs 6.60 ± 0.97, *p* ≤ 0.05; *n* = 10, Student’s *t*-test, Figure 4C).

Next, we attempted to improve the accuracy of functional analysis by performing a gait analysis. The instantaneous running speed of MCAO + ROSI mice was improved in comparison with MCAO mice 72 h after MCAO (64.33 ± 5.73 vs 75.58 ± 8.75, *p* ≤ 0.05; *n* = 6, Student’s *t*-test, Figure 4D). Moreover, MCAO + ROSI mice performed better in stride length (41.36 ± 10.37 vs 55.70 ± 7.34, *p* ≤ 0.05; *n* = 6, Student’s *t*-test, Figure 4E) and print area (24.52 ± 3.41 vs 32.86 ± 4.31, *p* ≤ 0.05; *n* = 6, Student’s *t*-test, Figure 4F) of the left front limb compared with MCAO mice 72 h after stroke, indicating an improvement in gait performance attributed to the inhibition of ACSL4.

The infarct volume of each group at 72 h after stroke was shown in Figure 4G. In agreement with the neurological deficit scores, compared with the MCAO group mice, those treated with rosiglitazone exhibited reduced infarct volume (48.76% ± 5.15 vs 32.10 ± 4.46, *p* ≤ 0.05; *n* = 5, Student’s *t*-test, Figure 4G).

We used lase speckle 2-dimensional imaging to detect the regional CBF at pre-, during and post-MCAO (Figure 4H). No significant differences were observed between the MCAO group and the MCAO + ROSI group at pre-, during, and post-MCAO, while indicated that the cerebral ischemia and reperfusion were similar in all animals.

### Inhibition of Acyl-CoA Synthetase Long-Chain Family Member 4 Suppresses Ferroptosis After Stroke

Previous studies have suggested that ACSL4 may be a target for the inhibition of ferroptosis; we explored the mechanism of ACSL4 in cerebral infarction in more detail.

**FIGURE 5:**
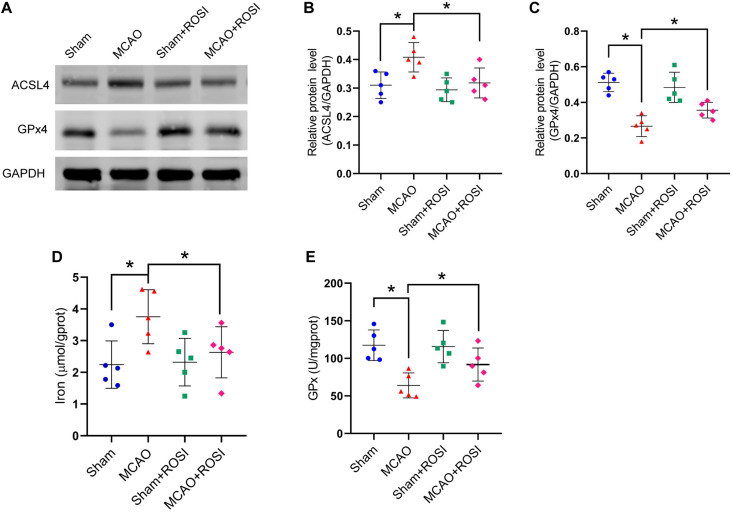
Inhibition of ACSL4 suppresses ferroptosis after stroke. **(A)** The protein levels of ACSL4 and GPx4 in peri-infarct region of sham group, MCAO group, sham + ROSI group and MCAO + ROSI group at 24 h after cerebral ischemic reperfusion, respectively. **(B,C)** Quantification of ACSL4 and GPx4 relative expression in mice. *P < 0.05, *n* = 5 each group. **(D,E)** Fresh tissue samples from the peri-infarct area of sham group, MCAO group, sham + ROSI group and MCAO + ROSI group at 24 h after stroke were collected to measure iron and GPx4 activity. *P < 0.05, *n* = 5 each group.

Compared with the MCAO group, MCAO + ROSI treatment significantly suppressed the increase in the protein level of ACSL4 in mice caused by stroke (MCAO group vs MCAO + ROSI group: 0.41 ± 0.05 vs 0.32 ± 0.05, *p* ≤ 0.05; *n* = 5, one-way ANOVA, Figures 5A,B). Compared with the MCAO group, MCAO + ROSI treatment significantly attenuated the decrease in the protein level of GPx4 in mice caused by stroke (0.27 ± 0.06 vs 0.36 ± 0.04, *p* ≤ 0.05; *n* = 5, one-way ANOVA, Figures 5A,C). The MCAO + ROSI group attenuated the iron accumulation caused by stroke, compared with the MCAO group (3.75 ± 0.85 vs 2.63 ± 0.81, *p* ≤ 0.05; *n* = 5, one-way ANOVA, Figure 5D). The GPx activity was significantly increased in the MCAO + ROSI group, compared with the MCAO group (63.99 ± 16.61 vs 91.80 ± 21.99, *p* ≤ 0.05; *n* = 5, one-way ANOVA, Figure 5E). The above results suggest that the inhibition of ACSL4 could suppress the occurrence of ferroptosis after cerebral infarction.

### Inhibition of Acyl-CoA Synthetase Long-Chain Family Member 4 Mitigate Lipid Peroxidation Related to Ferroptosis After Stroke

**FIGURE 6:**
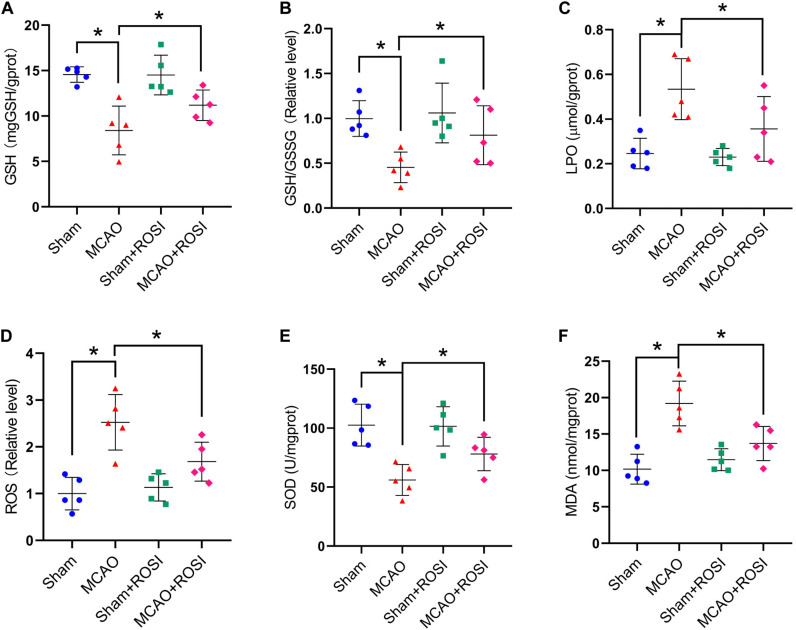
Inhibition of ACSL4 mitigate oxidative stress related to ferroptosis after stroke. Fresh tissue samples from the peri-infarct area of sham group, MCAO group, sham + ROSI group and MCAO + ROSI group at 24 h after stroke were collected to measure GSH level **(A)**, the GSH/GSSG ratio **(B)**, LPO level **(C)**, the ROS ratio **(D)**, SOD level **(E)**, and MDA level **(F)**. *P < 0.05, *n* = 5 each group.

Compared with the MCAO group, MCAO + ROSI treatment significantly attenuated the decrease in the levels of GSH and GSH/GSSG in mice caused by stroke (GSH: 8.41 ± 2.69 vs 11.18 ± 1.67, GSH/GSSG: 0.45 ± 0.17 vs 0.81 ± 0.33, *p* ≤ 0.05; *n* = 5, one-way ANOVA, Figures 6A,B).

Compared with the MCAO group, the levels of LPO and the relative level of ROS were significantly decreased in the MCAO + ROSI group (LPO: 0.53 ± 0.14 vs 0.36 ± 0.14, ROS: 2.52 ± 0.59 vs 1.68 ± 0.41, *p* ≤ 0.05; *n* = 5, one-way ANOVA, Figure 6C,D).

Compared with the MCAO group, MCAO + ROSI treatment significantly attenuated the decrease of SOD level and the increased MDA level in mice caused by stroke (SOD: 56.06 ± 13.16 vs 78.07 ± 14.09, MDA: 19.19 ± 3.08 vs 13.69 ± 2.35, *p* ≤ 0.05; *n* = 5, one-way ANOVA, Figures 6E,F). The above results indicate that inhibition of ACSL4 after stroke may reduce lipid peroxidation, which was considered to be related to the suppression of ferroptosis.

## Discussion

Ferroptosis is a novel mode of cell death that is caused by iron-dependent accumulation that is driven by lipid peroxidation, which differs significantly from apoptosis and autophagy in morphology, biochemistry and genetics (Dixon et al., 2012). Recent studies have shown that ferroptosis is an important form of cell death in neurodegenerative diseases, haemorrhagic stroke, brain trauma, intestinal ischemia and other diseases (Tuo et al., 2017; Chen B. et al., 2019; Li et al., 2019; Yan and Zhang, 2019). A recent study indicated that inhibition of ferroptosis protects mice from ischemia-reperfusion injury in a middle cerebral artery occlusion model, suggesting that ferroptosis leads to neuronal death after ischemic stroke (Tuo et al., 2017). It has been shown that reduced expression of glutathione peroxidase 4, which is essential for protection under hypoxic conditions, promotes ferroptosis (Becker et al., 2014; Gaschler et al., 2018). Iron accumulation can promote lipid peroxidation, which leads to GPx4 depletion and promotes ferroptosis production (Bogdan et al., 2016; Gaschler et al., 2018). Currently, GPx4 is often used as a molecular marker to identify cells that undergo ferroptosis *in vivo* or *in vitro*. In the present study, we investigated the underlying mechanism of ferroptosis in stroke. Our data showed that the ferroptosis biomarker GPx4 and COX2 both changed correspondingly after stroke. At the same time, the iron level in the brain tissue of the ischemic penumbra increased, indicating iron accumulation. Transmission electron microscopy is considered to be a sensitive and accurate method for monitoring ferroptosis *in vivo* and *in vitro*. The ultramicro-morphological features of TEM indicate cell membrane rupture and vacuolation, reduced mitochondrial volume, increased bilayer membrane density, reduced or absent mitochondrial cristae and normal nucleus size but a lack of chromatin condensation (Chen B. et al., 2019). We observed that the outer mitochondrial membrane of neurons had ruptured and the mitochondrial cristae had decreased or disappeared. Studies have shown that ferroptosis can be inhibited by iron chelators and several novel small molecules such as liproxstatin-1 and ferrostatin-1 (Bogdan et al., 2016). We used liproxstatin-1 to inhibit ferroptosis and found that changes in GPx4 and COX2 were inhibited after stroke, which further explained the occurrence of ferroptosis after stroke. Concurrently, it was found that inhibiting ferroptosis can reduce iron accumulation after stroke. Our results clarified that ferroptosis occurred after stroke.

Another feature of ferroptosis is the accumulation of lipid ROS (Zhang et al., 2020). In the GPx family, GPx4 is the only member that can block ROS-mediated lipid peroxidation (Xie et al., 2018). Therefore, the expression of GPX4 has been used as an important marker of ferroptosis. Furthermore, GSH, GSH/GSSG, GPx, SOD and MDA are important indicators for assessing the degree of lipid peroxidation; as such, they can help determine whether ferroptosis occurs in a stroke. Moreover, GSH is a predominant intracellular antioxidant sulfhydryl substance and plays an important role in antioxidant activity, protein sulfhydryl protection and amino acid transport across membranes. Reduced glutathione and total glutathione/oxidized glutathione are the main dynamic indicators of cellular redox status (Aquilano et al., 2014). Furthermore, GPx is a specific catalase for the hydrogen peroxide reduction of reduced glutathione and its activity represents the rate at which GSH is catalyzed (Xie et al., 2019). In this study, GPx activity, GSH levels, GSH/GSSG ratio, and SOD levels were decreased in stroke patients, but MDA levels and LPO levels were increased. At the same time, after the application of liproxstatin-1, a specific inhibitor of ferroptosis, the lipid peroxidation process was significantly blocked.

Recent studies have shown that ACSL4 expression may correspond to being a useful biomarker for monitoring ferroptosis (Yuan et al., 2016). The process of ferroptosis includes the preferential oxidation of the PE of AA and AdA. In enzymology, ACSL4 primarily esterifies CoA into free fatty acids in an adenosine triphosphate-dependent manner. Moreover, ACSL4 has a clear preference for long polyunsaturated fatty acids, such as AA and AdA. In addition, ACSL4-deficient cells are refractory to ferroptosis induced by GPx4 inactivation, indicating that ACSL4 may be a target for ferroptosis (Angeli et al., 2017; Doll et al., 2017). However, whether ACSL4 has a role in ferroptosis after stroke is still unclear. Neurological scoring and gait analysis have been widely used in clinical practice toassess the severity of stroke; in the present study, we assessed the severity of stroke by neurological deficits scores, neuroscore (28-point), gait analyses, and a corner test (Caballero-Garrido et al., 2017; Song et al., 2019). Instantaneous running speed, stride-length of the left front limb and print area of the left front limb were selected for gait analysis. We found that the 28-point neurological score, neurological deficit score, corner test and gait analysis were all affected by MCAO. Importantly, our results show that after the application of rosiglitazone to inhibit ASCL4, the brain damage of mice suffering from MCAO was significantly improved compared with the control mice. It can be concluded that the inhibition of ACSL4 promotes the recovery of neurological function after stroke and ASCL4 may be the target of stroke. Indeed, thiazolidinediones (TZNs) have been reported to specifically inhibit ACSL4 compared with other ACSL isoforms. Thiazolidinediones act as a peroxisome proliferator-activated receptor g (PPARg) agonists and are currently being developed and marketed as insulin sensitizers; however, TZNs have been shown to act independently of PPARg on ferroptosis, which is attributed to their inhibitory effect on ACSL4 (Askari et al., 2007; Kan et al., 2014; Doll et al., 2017; Wei et al., 2020). Among them, rosiglitazone (a TZN-like compound), which is used to treat animals, can specifically inhibit ACSL4 to exert corresponding effects. In the present experiment, the application of rosiglitazone significantly inhibited changes in GPX4, the signature protein of ferroptosis following cerebral ischemia. Concurrently, it reduced iron accumulation after stroke. The above results suggest that ACSL4 plays an important role in the development of ferroptosis after stroke and that the inhibition of ACSL4 can suppress ferroptosis. We also wanted to review whether ACSL4 had the same effect on the accumulation of lipid ROS in ferroptosis after stroke. We assayed GSH, GSH/GSSG, GPx, SOD and MDA, which are important markers in lipid peroxidation and found that rosiglitazone (an ACSL4-specific inhibitor) inhibited a decrease in GPx activity, GSH levels and GSH/GSSG ratio after cerebral ischemia, indicating its inhibitory effect on lipid peroxidation. Moreover, rosiglitazone inhibited a decrease in SOD and an increase in MDA and LPO. Most importantly, the accumulation of lipid ROS in cerebral ischemia was significantly reduced after the application of rosiglitazone. Indeed, after the application of rosiglitazone, the lipid peroxidation process was significantly blocked. Whether ACSL can promote the neurological recovery of stroke through mechanisms other than the ferroptosis pathway is still unknown; this will be the focus of our future research.

Ferroptosis and apoptosis are two distinct mechanisms of neuronal death. Ferroptosis is a form of necrosis depending on irons that is regulated by cellular stresses (Fricker et al., 2018). Erastin-induced ferroptosis is lack of typical characteristics of cell death such as nuclear shrinkage and swelling of organelles (Dixon et al., 2012). The generation of ferroptosis relies on peroxidation of lipids, in which mitochondrial activity and abnormal nuclear translocation may play a role (Seiler et al., 2008). Apoptosis is the classical programmed cell death pathway, which can induce by both internal and external signals such as death receptors (Micheau and Tschopp, 2003). Therefore, our study provides a novel perspective for the recovery after stroke.

## Conclusion

**FIGURE 7:**
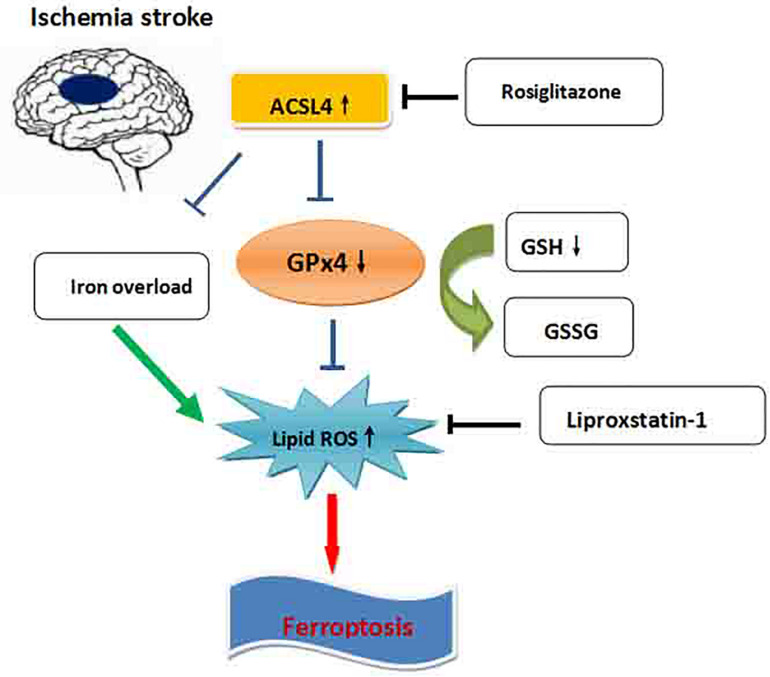
Schematic overview of ferroptosis in ischemic stroke. The iron overload in ischemic stroke increases the lipid ROS, which leads to ferroptosis. ACSL4 promotes ferroptosis through blocking the GPx4, which inhibits the lipid ROS elevation. Blocking the ACSL4 by rosiglitazone significantly promotes the recovery of neurological deficits after ischemic stroke by inhibition of ferroptosis.

Our study found that ferroptosis is an important form of cell death in instances of ischemia stroke. Furthermore, ACSL4 is an important target of ferroptosis, and the inhibition of ACSL4 promotes neurological recovery after stroke by regulating ferroptosis (Figure 7).

## Data Availability Statement

The original contributions presented in the study are included in the article/supplementary material, further inquiries can be directed to the corresponding author/s.

## Ethics Statement

All experimental procedures were approved by the Committee of Experimental Animals of Hebei Medical University, Shijiazhuang, China, and conducted in accordance with the National Institutes of Health guidelines.

## Author Contributions

JC and XW: conception and design of the research. LY and LG: acquisition of data. JH: analysis and interpretation of the data. LC and JZ: statistical analysis. JC and QS: writing of the manuscript. XW: critical revision of the manuscript for intellectual content. All authors contributed to the article and approved the submitted version.

## Conflict of Interest

The authors declare that the research was conducted in the absence of any commercial or financial relationships that could be construed as a potential conflict of interest.

## Abbreviations

ACSL4: Acyl-CoA synthetase long-chain family member 4
COX2: cyclooxygenase 2
GPx4: glutathione peroxidase 4
GSH: reduced glutathione
GSH/GSSG: total glutathione/oxidized glutathione
LPO: lipid peroxidation
MCAO: middle cerebral artery occlusion
MDA: malonaldehyde
ROS: reactive oxygen species
ROSI: rosiglitazone
SOD: superoxide.
